# Development of Micro-Heaters with Optimized Temperature Compensation Design for Gas Sensors

**DOI:** 10.3390/s110302580

**Published:** 2011-03-01

**Authors:** Woo-Jin Hwang, Kyu-Sik Shin, Ji-Hyoung Roh, Dae-Sung Lee, Sung-Hoon Choa

**Affiliations:** 1 Graduate School of NID Fusion Technology, Seoul National University of Science and Technology, Gongneung-Dong, Nowon-Gu, Seoul 139-743, Korea; E-Mail: hwj3237@naver.com (W.-J.H.); 2 Convergence Sensor & Device Research Center, Korean Electronics Technology Institute, Yatap-dong, Bundang-gu Seongnam, Gyeonggi Province 463-816, Korea; E-Mails: neokarion@keti.re.kr (K.-S.S.); jhr726@korea.ac.kr (J.-H.R.)

**Keywords:** micro-heater, micro-hotplate, NDIR gas sensor, semiconductor gas sensor, uniform heating characteristics, infrared radiation

## Abstract

One of the key components of a chemical gas sensor is a MEMS micro-heater. Micro-heaters are used in both semiconductor gas sensors and NDIR gas sensors; however they each require different heat dissipation characteristics. For the semiconductor gas sensors, a uniform temperature is required over a wide area of the heater. On the other hand, for the NDIR gas sensor, the micro-heater needs high levels of infrared radiation in order to increase sensitivity. In this study, a novel design of a poly-Si micro-heater is proposed to improve the uniformity of heat dissipation on the heating plate. Temperature uniformity of the micro-heater is achieved by compensating for the variation in power consumption around the perimeter of the heater. With the power compensated design, the uniform heating area is increased by 2.5 times and the average temperature goes up by 40 °C. Therefore, this power compensated micro-heater design is suitable for a semiconductor gas sensor. Meanwhile, the poly-Si micro-heater without compensation shows a higher level of infrared radiation under equal power consumption conditions. This indicates that the micro-heater without compensation is more suitable for a NDIR gas sensor. Furthermore, the micro-heater shows a short response time of less than 20ms, indicating a very high efficiency of pulse driving.

## 1. Introduction

The number of applications for MEMS micro-heater devices is increasing rapidly, as they are key components in subminiature micro-sensors such as wind sensors [1], humidity sensors [2] and gas sensors. A MEMS micro-heater that emits heat by applying a current to a resistor has the advantage for low power driving as well as a very short response time. In particular, these days MEMS micro-heaters are becoming increasingly important in portable electronics applications where low-voltage and low-power designs are required. Several researches have been conducted on micro-heaters that use SiC [3], Pt [1,4–6], poly-Si [7], single crystal silicon [8], and TiN [9] as the heating layer. A study of temperature distribution uniformity over the heating plate through control of the heat distribution was previously conducted [10]. Polyimide [4] and SOI [11] have also been studied for use as membrane materials.

In a semiconductor gas sensor [4–6,12,13], a micro-heater is used as a hot plate which controls the temperature of the sensing layer. The semiconductor gas sensor utilizes semiconductor properties of surface adsorption to detect changes in resistance as a function of varying concentrations of different gases. In order to detect these resistive changes, the heater temperature must be held constant and uniform over the heater area. Therefore, sensitivity, selectivity and response time of the semiconductor gas sensor are dependent on the sensing layer material and operating temperature of the micro-heater [12]. Use of a micro-heater is necessary in most gas sensors because the gas chemical reaction in the sensing layer takes places at high temperatures. Furthermore, a micro-heater can be used as the infrared source in a non-dispersive infrared (NDIR) gas sensor [3,10,14,15]. The micro-heater is used to radiate infrared radiation to make the gas molecules excited inside the gas sensing chamber. Generating higher levels of heat from the micro-heater is a very important factor in improving the sensitivity of the NDIR sensor [10,15] since infrared absorption of gas in the NDIR gas sensing chamber increases with increasing infrared radiation through the micro-heater. In addition, nowadays much interest is being paid to miniaturization and low power driving of sensors. A micro-heater that is capable of low power driving [11] needs to be developed in particular for the NDIR sensor. Therefore, the temperature and infrared radiation characteristics of different micro-heaters should be considered for the condition of equal power consumption.

Micro-heaters can be used in both semiconductor gas sensors and NDIR gas sensors, but they require different heat dissipation characteristics depending on the use. For semiconductor gas sensors, sensitivity and selectivity are dependent on the heating temperature of the heater and a uniform temperature is required over a wide area of the heater. On the other hand, for the NDIR gas sensor, the micro-heater needs high levels of infrared radiation in order to increase the sensitivity of the sensor. In this study, a novel micro-heater design that improves the uniformity of heat dissipation on the heating plate by varying the power dissipation over the heater is proposed. In addition, as a heating element, a thin film-type poly-Si material was selected, which has a high reliability at temperatures of more than 400 °C, and a wide bandwidth of wavelengths from 2 to 6 μm to ensure that it can be used as a semiconductor gas sensor as well as a NDIR gas sensor.

Using a thermal imaging camera and infrared detector, the heat distribution and infrared radiation level of the micro-heater both with and without the power distribution compensation were measured for comparison. In this way, the feasibility as to whether the micro-heater was suitable for use as both a semiconductor gas sensor and NDIR gas sensor was examined.

## 2. Design and Fabrication of the Micro-Heater

### 2.1. Design of Micro-Heater for Compensating the Power Consumption

**Figure 1.**
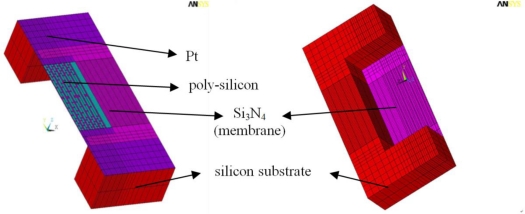
Numerical mesh model for the MEMS micro-heater.

The 3D finite element method (FEM) was used to estimate the temperature distribution on the heating plate. The commercial software ANSYS 11 was used as the finite element modeling tool. A thermal-electric coupled field analysis was also performed to observe the heat release and heat distribution of the micro-heater as current was applied. Figure 1 shows the model of the micro-heater used for this analysis. The total size of the sensor was 2.4 × 2.6 mm, which is equal to that of the silicon die, and the thickness of the silicon die was 525 μm. A poly-Si film was used as the heating element of size 1.1 × 1.1 mm and thickness of 2,000 Å. The poly-Si film was supported by a Si_3_N_4_ membrane of size 1.4 × 1.6 mm and was designed to be very thin, 1 μm, in order to minimize heat loss due to thermal conduction. As an electrical contact, Pt with a thickness of 2,000 Å was used. An eight-node tetrahedral element was selected for the numerical modeling of each part, applying a more dense mesh for the poly-Si film and membrane (Si_3_N_4_) to enhance the accuracy of the analysis. The material properties data used for the FEM thermal simulation are shown in Table 1. The emissivity of the poly-Si used as the heating element is known to be 0.7, and so this value was used in the analysis. An air convection coefficient of 5 Wm^−2^ K^−1^ was used and the radiation constant was defined as the Stefan-Boltzmann constant 5.67 × 10^8^ Wm^−2^ K^−4^.

In a conventional micro-heater, the temperature of the membrane tends to decrease dramatically away from the center of the membrane. The reason for this tendency is the greater heat loss resulting at around the outer areas of the heater. That is, the heat produced from the poly-Si resistor (or heater) on the thin Si_3_N_4_ membrane with its low thermal conductivity flows out to the thick silicon substrate with its high thermal conductivity located at the edge of the membrane. In the case of a micro-heater used in a semiconductor gas sensor, a locally concentrated high temperature in the membrane center could undermine the sensitivity and selectivity of the sensor. In order to prevent such heat loss, it is necessary to increase the generation of heat at the edge of the heater membrane, where heat loss is much larger than at the center of the poly-Si resistor. To achieve this increase in heat generation it is necessary to increase the power consumption at the edge of the membrane where the majority of heat is lost.

**Figure 2.**
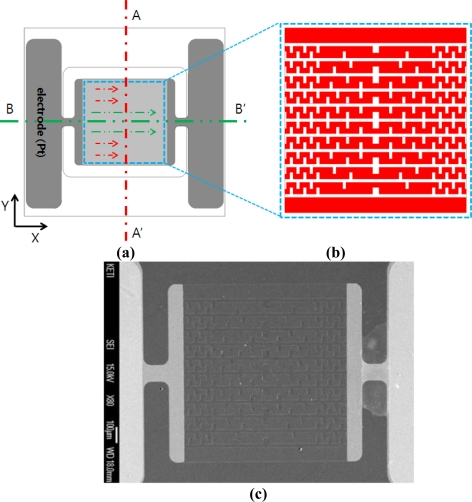
The power compensation structure of poly-Si film to enhance the temperature distribution. **(a)** current flow map in the micro-heater. **(b)** schematic drawing of compensation design of poly-Si film. **(c)** picture of compensation design in poly-Si micro-heater.

Figure 2 shows a schematic drawing of the design proposed to improve the uniformity of the heat dissipation across the heating plate. Since the heat loss of the micro-heater takes place in both the x and y directions, compensation should be considered in both directions. In Figure 2(a), there is no potential difference along the A-A′ line (or in the y-direction) as indicated by the arrows. Since the voltage is constant, from Equation (1):

(1) $$P=\frac{V^{2}}{R}$$

The power consumption applied is inversely proportional to resistance. Therefore, a reduction in resistance in the outside area of the micro-heater will lead to an increase in power consumption; subsequently increasing the heating temperature. Therefore, in the y-direction, the outer area is designed to have a coarser and larger pattern of poly-Si than at the center in order to decrease resistance and hence temperature.

Along the line B-B′ (or in the x-direction) in Figure 2(a), the electrode and the heating plate are connected in series. Therefore, the current flowing along the line B-B′ (in the direction of the arrows) is equal. Since the current is constant, from Equation (2):

(2) $$P=I^{2}\times R$$

The power consumption is proportional to the resistance. Therefore the heating temperature of the outside area of the micro-heater can be increased by increasing the resistance of the outer area of the poly-Si heater to compensate for the heat loss in the vicinity of the silicon substrate. Therefore, in the x-direction, the outside area has a smaller and denser pattern of poly-Si than at the center area in order to increase the resistance and hence temperature.

**Figure 3.**
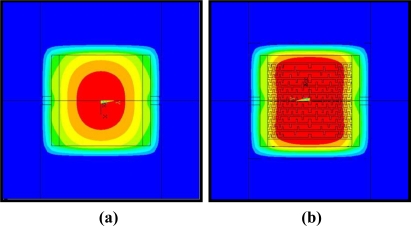
Analyzed heating characteristics of micro-heaters by FEM simulation. **(a)** standard poly-Si micro-heater. **(b)** power compensated poly-Si micro-heater.

Figure 2(b) shows the configuration of the power compensated micro-heater for improved temperature uniformity in both x and y directions. Figure 2(c) shows an SEM picture of the fabricated power compensated poly-Si micro-heater. Figure 3(a) shows simulation results for the thermal distribution of the standard poly-Si micro-heater. The red color indicates the area over which the temperature reaches 90% of the maximum temperature. These results confirm that the high temperature area is concentrated at the center of the micro-heater. Figure 3(b) shows the simulation results for the thermal distribution of the power compensated micro-heater. The temperature distribution of the micro-heater is uniform, and the area covered by 90% of the maximum temperature on the heating plate has increased by more than 2.5 times in comparison to the standard micro-heater. This indicates that the new design is effective in improving the uniformity of the heat distribution over the heating plate.

### 2.2. Fabrication of Micro-Heater

**Figure 4.**
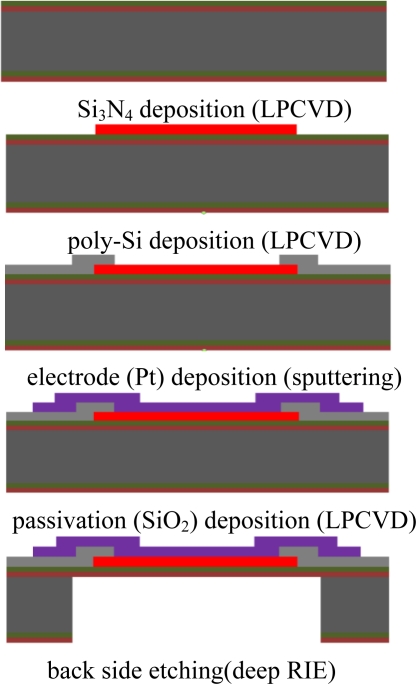
The fabrication process of the MEMS micro-heater.

A micro-heater was fabricated based on the simulation results. Figure 4 shows the fabrication process used to manufacture the micro-heater. The micro-heater was 2.4 × 2.6 mm, and the size of the membrane was 1.2 × 1.4 mm. In order to fabricate the micro-heater using poly-Si as the heating element, a low stress silicon nitride film 1 μm in thickness was deposited on the silicon wafer (100) using the low pressure chemical vapor deposition (LPCVD) process. The poly-Si thin film 2000 Å thick was then deposited on the nitride film also using the LPCVD process. In order to control the resistance of the poly-Si, phosphorous doping was applied using the ion implantation process. The sheet resistance of the poly-Si film was approximately 200 Ohm/square measured with a 4-point probe. The power compensated design was fabricated by etching areas of the poly-Si using the reactive ion etching (RIE) process. The Pt for the electrical contact was deposited at a thickness of 2,000 Å using the sputtering process. In order to prevent oxidation of the poly-Si during heating, an SiO_2_ passivation layer was deposited with a thickness of 2,000 Å using plasma enhanced chemical vapor deposition (PECVD), followed by partial etching using the buffered oxide etchant (BOE). The membrane was fabricated by anisotropic etching of the Si substrate using the deep RIE process.

**Figure 5.**
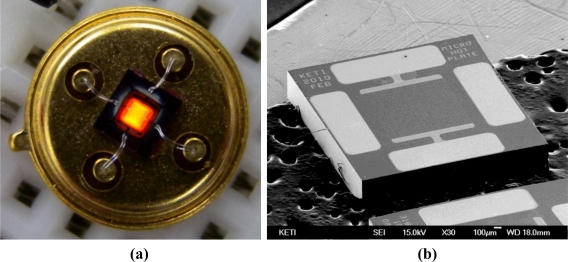
Fabricated micro-heater. **(a)** packaged micro-heater using TO39 package. **(b)** SEM image of the micro-heater.

Figure 5 shows the as fabricated micro-heater. It was found that a membrane with a thickness of 1 μm was properly fabricated in the center of the device and that the heating element made of poly-Si was also properly formed on the membrane. As a final step, the device was packaged in a TO-39 package and an electrical contact was made by wire bonding.

## 3. Results and Discussion

### 3.1. Measurement of Temperature Uniformity on the Heating Plate of the Micro-Heater

The heating characteristics of the micro-heater were measured with a thermal imaging camera (SC-5500) from FLIR Systems. The measurement range of the infrared spectrum of the thermal imaging camera is in the wavelength region between 2 and 12 μm and the camera can measure temperatures above 1,000 °C.

**Figure 6.**
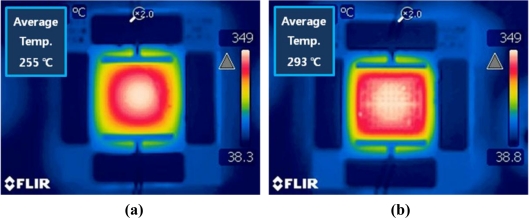
Measured average temperature of micro-heaters at 350 °C as maximum temperature. **(a)** standard poly-Si micro-heater. **(b)** power compensated poly-Si micro-heater.

**Figure 7.**
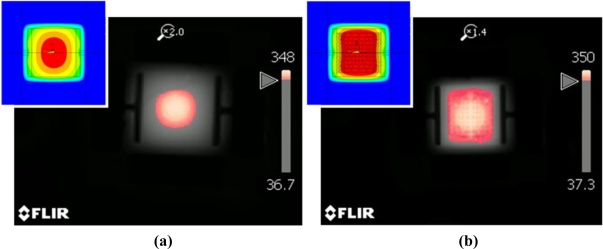
Measured within 10% of high temperature distribution of micro-heaters at 350 °C as maximum temperature. **(a)** standard poly-Si micro-heater. **(b)** power compensated poly-Si micro-heater.

The uniformity of the heating characteristics of the micro-heater can be evaluated by measuring the average temperature and the size of the area of >90% of the maximum temperature. Figure 6 was taken using the thermal imaging camera, and shows the measurement results for the average temperatures of the micro-heaters. There is a substantial difference in the average temperature recorded between the two micro-heaters with and without the power compensation. In order to objectively compare the average temperatures, only the heat over the area of the membrane was considered. Each micro-heater emitted heat at a maximum temperature of around 350 °C however the power compensated design shows an average temperature of around 293 °C, which is around 40 °C higher than that obtained from the standard heater. In Figure 7, the red color indicates the area at which the temperature reaches 90% of the maximum temperature. In comparison to the results of the numerical analysis, shown in Figure 3, the practical results show a high level of agreement. As seen in the simulation results the power compensated micro-heater has an area of heat exceeding 90% of the maximum temperature 2.5 times that of the standard micro-heater. Furthermore, for the power compensated micro-heater, the area covered by temperatures exceeding 90% of the highest temperature became more than 80% of the total heating area. These results show that the power compensated micro-heater is more suitable for use as a heating element for a temperature-dependent semiconductor gas sensor than a standard micro-heater.

### 3.2. Measurement of Temperature Characteristics and Infrared Radiation for Equal Power Consumption

**Figure 8.**
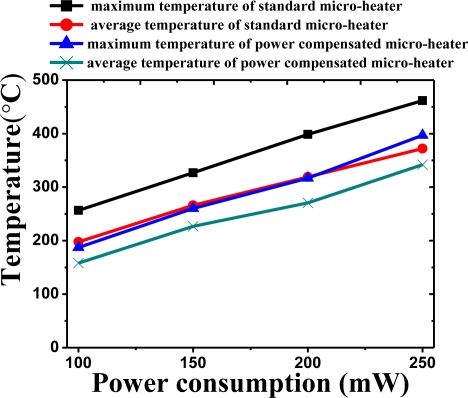
The measured maximum and average temperature at different power consumption of micro-heaters with standard micro-heater and power compensated micro-heater.

**Figure 9.**
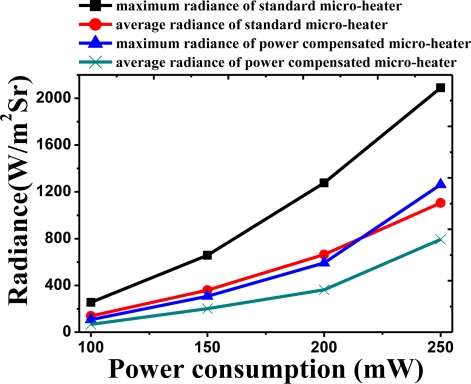
The measured maximum and average radiance at different power consumption of micro-heaters with standard micro-heater and power compensated micro-heater.

Figure 8 shows the maximum temperature and average temperature of the two micro-heaters when operated with equal levels of power consumption. As power consumption was varied from 100 mW to 250 mW, the thermal characteristics of the two micro-heaters was measured. Both the maximum and average temperatures decrease for the micro-heater with power compensation for equal levels of power consumption. When a power consumption of 250 mW was applied, the maximum temperature of the standard micro-heater was measured to be around 460 °C with an average temperature of around 370 °C. In the case of the power compensated micro-heater, the maximum temperature was approximately 400 °C, 60 °C lower than that of the standard micro-heater. In addition, the average temperature was measured to be 340 °C, around 30 °C lower. The results of the radiation measurement also showed a similar trend. Figure 9 shows the maximum and average radiance values. The radiation of the standard micro-heater was measured to be 2,100 Wm^−2^Sr^−1^ around two times higher than that of the power compensated micro-heater at 1,100 Wm^−2^Sr^−1^. It is thought that the lower level of infrared radiation is due to extra power being channeled into compensating for the heat loss in the vicinity of the silicon substrate. This indicates that the standard micro-heater is more efficient as an infrared source in a NDIR gas sensor.

### 3.3. Evaluation of Response Characteristics of Micro-Heater

**Figure 10.**
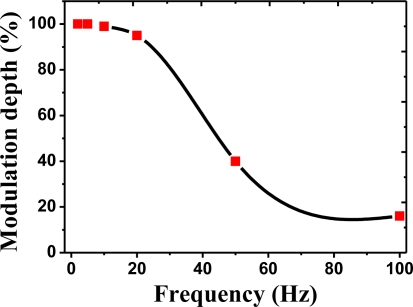
The modulation depth of the micro-heater ranging from frequency of 0∼100 Hz.

The modulation depth in the micro-heater is a very important factor when pulse type infrared radiation is required for the NDIR gas sensor. To obtain high detecting accuracy, it is necessary to develop efficient and modulating IR sources. For example, the pyroelectric IR sensor is only activated when the infrared radiation varies. Therefore, a NDIR gas sensor often needs a mechanical chopper or chopper control circuits. However, if a pulse-type infrared source with a high modulation depth is used, devices such as choppers become unnecessary. In addition, the pulse driving method can reduce the power consumption of the sensor. The response characteristics and modulation depth of the standard micro-heater was measured. A thermal imaging camera (SC-5500) manufactured by FLIR Systems was utilized to measure the response speed. The rise time of the micro-heater was measured to be less than 20 ms, indicating a high enough speed to replace a mechanical chopper. Figure 10 shows the modulation depth of the standard micro-heater. The modulation depth at 10 Hz was 99%, a negligible change. At 20 Hz, the modulation depth was 95%, indicating reasonably stable infrared rays could be radiated when pulse-type driving is used. At 50 Hz, measurements showed that around 44% of the infrared rays were radiated. As a result, it is expected that a very high efficiency can be obtained in the case of pulse driving.

## 4. Conclusions

In this study, a poly-Si thin film was used as the heating element for two micro-heater designs for use in semiconductor and NDIR gas sensors. Through numerical modeling, a novel micro-heater was designed and fabricated aimed at improving the uniformity of heat dissipation on the heating plate by compensating for the variation in power consumption over the heater. It was found that the uniform area of high temperature for the power compensated micro-heater was around 2.5 times larger than that of the uncompensated micro-heater. In this case, the area where the temperature was more than 90% of the maximum temperature became more than 80% of the total heating area. This indicates that the heating area was substantially increased and shows that the micro-heater with the compensation design is suitable for use in a temperature-dependent semiconductor gas sensor. Meanwhile, the uncompensated micro-heater showed higher levels of infrared radiation when equal levels of power consumption were applied to the two micro-heaters. At a power consumption of 250 mW, the infrared radiation was around two times higher than that produced by the power compensated micro-heater, which is due to the extra power used to compensate for the heat loss in the vicinity of the silicon substrate. Therefore it is recommended that the uncompensated micro-heater is more suitable as an infrared source in a NDIR gas sensor. In addition, the micro-heater fabricated with a poly-Si thin film in this study shows a short response time, less than 20 ms, indicating a speed high enough to replace a mechanical chopper.

## Figures and Tables

**Table 1.** Material properties of a micro-heater used in FEM thermal simulation.

	**Silicon**	**Polysilicon**	**Silicon nitride**	**Platinum**	**Units**

**Electrical resistivity**	—	4 × 10^−4^		1.06 × 10^−7^	Ohm-m
**Thermal conductivity**	157	34	4.5	71	W/mK
**Density**	2320	1800	3100	21450	kg/m^3^
**Thermal capacity**	702	170	700	134	J/kgK
